# Foetal programming by methyl donor deficiency produces steato-hepatitis in rats exposed to high fat diet

**DOI:** 10.1038/srep37207

**Published:** 2016-11-17

**Authors:** Anaïs Bison, Aude Marchal-Bressenot, Zhen Li, Ilef Elamouri, Eva Feigerlova, Lu Peng, Remi Houlgatte, Bernard Beck, Gregory Pourié, Jean-Marc Alberto, Remy Umoret, Guillaume Conroy, Jean-Pierre Bronowicki, Jean-Louis Guéant, Rosa-Maria Guéant-Rodriguez

**Affiliations:** 1 Inserm U954, Nutrition-Genetics-Environmental Risk Exposure (N-GERE), University of Lorraine, BP 184, 54511, Vandœuvre-lès-Nancy, France

## Abstract

Non-alcoholic steatohepatitis (NASH) is a manifestation of metabolic syndrome, which emerges as a major public health problem. Deficiency in methyl donors (folate and vitamin B12) during gestation and lactation is frequent in humans and produces foetal programming effects of metabolic syndrome, with small birth weight and liver steatosis at day 21 (d21), in rat pups. We investigated the effects of fetal programming on liver of rats born from deficient mothers (iMDD) and subsequently subjected to normal diet after d21 and high fat diet (HF) after d50. We observed increased abdominal fat, ASAT/ALAT ratio and angiotensin blood level, but no histological liver abnormality in d50 iMDD rats. In contrast, d185 iMDD/HF animals had hallmarks of steato-hepatitis, with increased markers of inflammation and fibrosis (caspase1, cleaved IL-1β, α1(I) and α2(I) collagens and α-SMA), insulin resistance (HOMA-IR and Glut 2) and expression of genes involved in stellate cell stimulation and remodelling and key genes triggering NASH pathomechanisms (transforming growth factor beta super family, angiotensin and angiotensin receptor type 1). Our data showed a foetal programming effect of MDD on liver inflammation and fibrosis, which suggests investigating whether MDD during pregnancy is a risk factor of NASH in populations subsequently exposed to HF diet.

Non-alcoholic fatty liver disease (NAFLD) is a major consequence of central obesity and metabolic syndrome, with a spectrum that ranges from simple steatosis to steatohepatitis (NASH). The metabolic syndrome is a cluster of components, which includes abdominal obesity, high triglycerides and insulin resistance. NASH is considered as a visceral manifestation of metabolic syndrome, which emerges as one of the major public health problem, in the context of the epidemic of severe obesity in Western countries^1^. It results from complex mechanisms, which trigger steatosis, inflammation, cellular stress and remodelling of liver tissue^2^. The concept of developmental origins of health and disease (DOHaD) considers that the foetal programming during pregnancy and early post-natal life has a long-term impact on the risk of central obesity and other components of metabolic syndrome. This concept is now evidenced by many experimental and epidemiological studies^2^. However, little is known on the influence of nutritional conditions during pregnancy and the subsequent post-natal risk of NAFLD and more specifically of NASH.

Over the past decade, epidemiological and experimental studies have clearly demonstrated an association between nutritional metabolites of the one carbon metabolism (1-CM) and manifestations of foetal programming^3^. Cellular methionine originates from the remethylation pathway of homocysteine by methionine synthase, which uses vitamin B12 (methyl-cobalamin) as a cofactor. The deficit in methyl donors, in particular folate and vitamin B12 leads to decreased synthesis of S-adenosylmethionine (SAM), the methyl donor involved in methylation of DNA and proteins, which regulate gene expression, including histones, nuclear receptors and their co-regulators^3^.

Recent large epidemiological studies have shown that 1-CM markers and methyl donor deficiency (MDD) during pregnancy influence the risk of small birth weight and subsequent events related to foetal programming, including metabolic syndrome and insulin resistance. Studies in India and Nepal found vitamin B12 deficiency during pregnancy to be the most significant predictor of small birth weight, abdominal fat and insulin resistance in children aged 6 to 8 years^4^,^5^,^6^. A variant of SAM decarboxylase was associated with childhood obesity, in another Indian study^7^. In addition, the rates of methylation of homocysteine and transmethylation of methionine were significantly lower in 15 subjects with NASH compared with 19 healthy age-matched controls^8^. Experimental models have also highlighted the influence of methyl donors, vitamin B12 and folate on foetal programming and related components of metabolic syndrome. We showed that deficiency in folate and vitamin B12 during gestation and lactation of rat dams produces small birth weight, increased central fat mass, liver steatosis and cardiac hypertrophy in pups^9^,^10^. The liver steatosis resulted from impaired fatty acid β-oxidation and impaired energy metabolism. It was reversible when animals were fed subsequently a normal diet. The underlying molecular mechanisms were linked to the hypomethylation of PGC1-α and the subsequent dysregulation of its interaction with nuclear receptors, ERα, PPARs, ERRα and HNF-4. Consistently, another study showed that the lack of PPAR-α enhanced steatohepatitis, while PPAR-α agonist had the opposite effect, in a transgenic mice model^11^. In addition, a study of liver biopsies from NAFLD patients showed that the methylation of PGC1-α promoter was linked to HOMA-IR and plasma fasting insulin levels^12^. Recently, we analyzed the profile of the liver transcriptome and methylome of 21-day pups from rat dams with deficiency in folate and vitamin B12 during gestation and lactation^13^. Changes in both gene expression and DNA methylation were limited to 266 genes, most of which participated in pathomechanisms of NASH. It is noticeable that methyl donor supplementation protects from liver steatosis in rats fed a high fat-sucrose diet, in contrast to the risk associated with MDD^14^.

In summary, MDD during pregnancy predicts central obesity and insulin resistance in children and produces liver steatosis through dysregulations of fatty acid oxidation in pups from rat females subjected to deficiency during gestation and lactation. Whether a foetal programming effect of MDD influences the subsequent risk of NASH remains unknown. In this study, we evaluated the foetal programming effects of MDD on the risk of NASH in rats subjected subsequently to normal diet during the post-weaning period and high-fat (HF) diet between day (D)50 and D185 of adult life.

## Results

### Rats born from dams with MDD diet during pregnancy and lactation and subjected to HF diet after D50 had increased abdominal fat and ASAT/ALAT ratio at D50 and biochemical and histological hallmarks of NASH at D185

**Figure 1:**
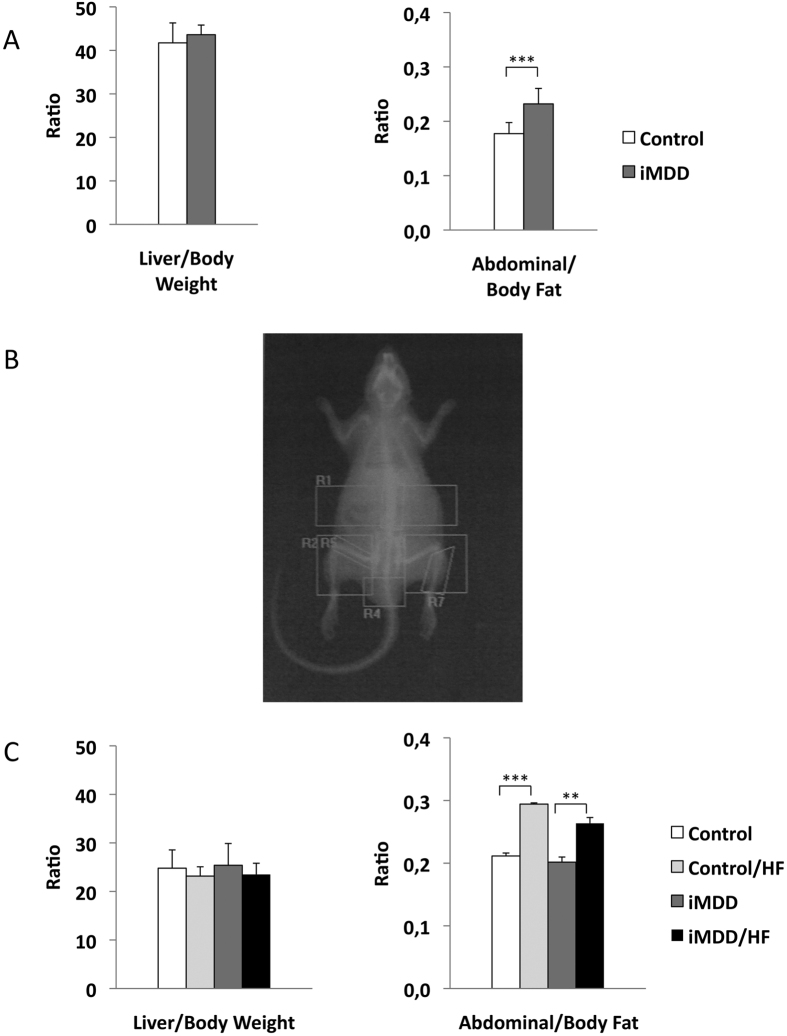
Biophysical features of D50 and D 185 rats. D50 rats were born from dams fed a methyl donor deficient diet (iMDD) diet during pregnancy and lactation and subsequently subjected to high fat (HF) diet between D50 and D185. (**A**) Body weight/liver weight ratio was similar in iMDD, compared to control D50 animals. In contrast, the ratio of abdominal/body fat determined by DEXA was greater. (**B)** Example of DEXA examination in an iMDD animal. (**C**) The liver weight/body weight ratio were similar in iMDD/HF, compared to control D185 animals. In contrast, the ratio of abdominal/body fat determined by DEXA was greater, as observed in D50 animals. Data are reported as means ± SD (n = 8 in each group), *p < 0.05, **p < 0.01, ***p < 0.001.

**Figure 2:**
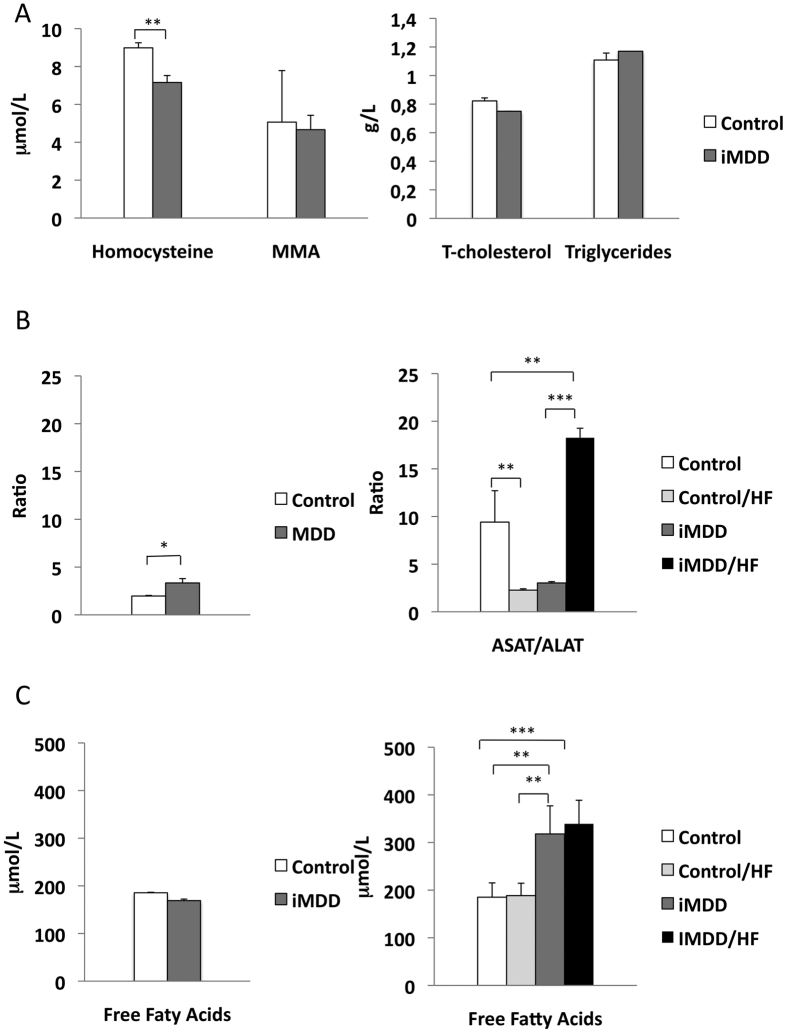
Biochemical parameters of D50 and D 185 rats. D50 rats were born from dams fed a methyl donor deficient diet (iMDD) during pregnancy and lactation and subsequently subjected to high fat (HF) diet between D50 and D185. (**A**) Plasma concentrations of methylmalonic acid (MMA), homocysteine, triglycerides and cholesterol of iMDD rats were similar to those of control animals. (**B**) ASAT/ALAT ratio of iMDD D50 and iMDD/HF D185 rats were increased, compared to the respective control animals. (**C**) Free fatty acids concentration among groups was similar at D50. At D185 the free fatty acid concentration was higher in iMDD and iMDD/HF groups, compared to their respective control groups. Data are reported as means ± SD (n = 8 in each group), *p < 0.05, **p < 0.01, ***p < 0.001.

**Figure 3:**
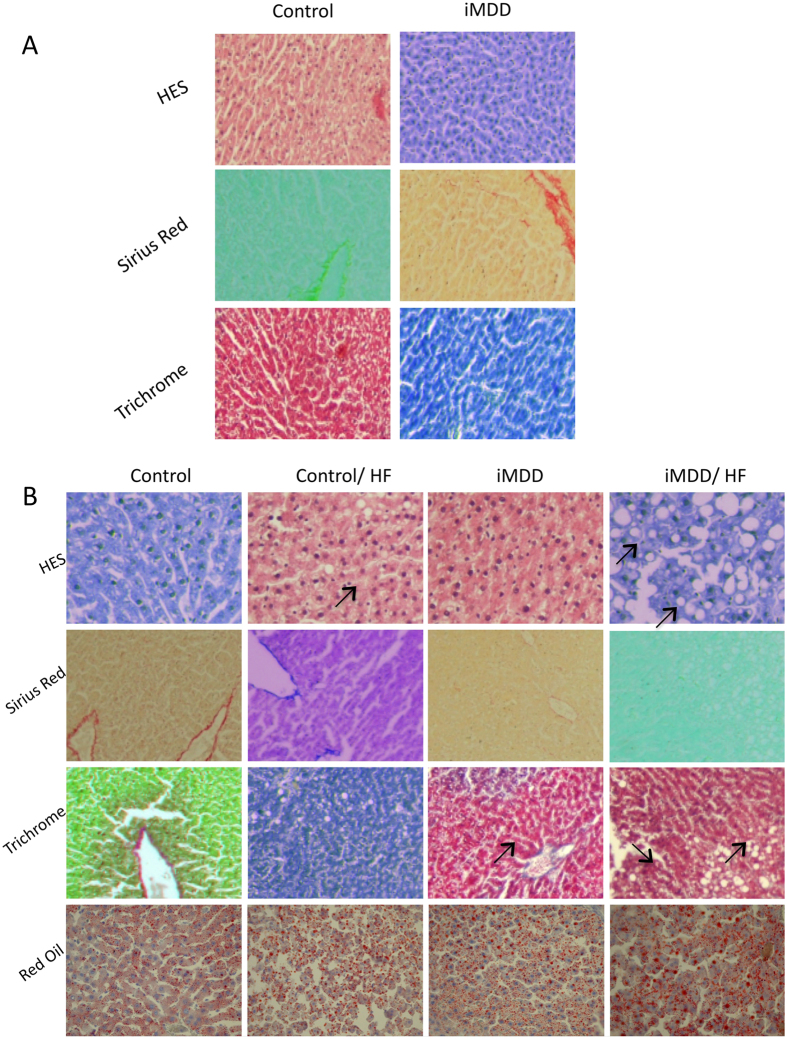
Liver histology of D50 and D 185 rats. D50 rats were born from dams fed a methyl donor deficient diet (iMDD) diet during pregnancy and lactation and subsequently subjected to high fat (HF) diet between D50 and D185. (**A**) Histological analysis in liver from D50 iMDD and control animals, with Hematoxylin Eosine (HES), Sirius Red staining (specific for collagen and reticulin fibers) and Masson Trichrome staining (specific for collagen fibers). Neither microvesicular steatosis nor fibrosis was detected in iMDD and control animals. (**B**) Histological analysis in liver from D185 iMDD/HF animals, with Hematoxylin Eosine (HES), Sirius Red staining (specific for collagen and reticulin fibers), Masson Trichrome staining (specific for collagen fibers) and red oil (for visualization of fat cells). We observed a dramatic microvesicular steatosis and fibrosis (indicated by arrows) in tissue samples from iMDD/HF rats, and a much weaker steatosis in control/HF and iMDD rats.

**Figure 4:**
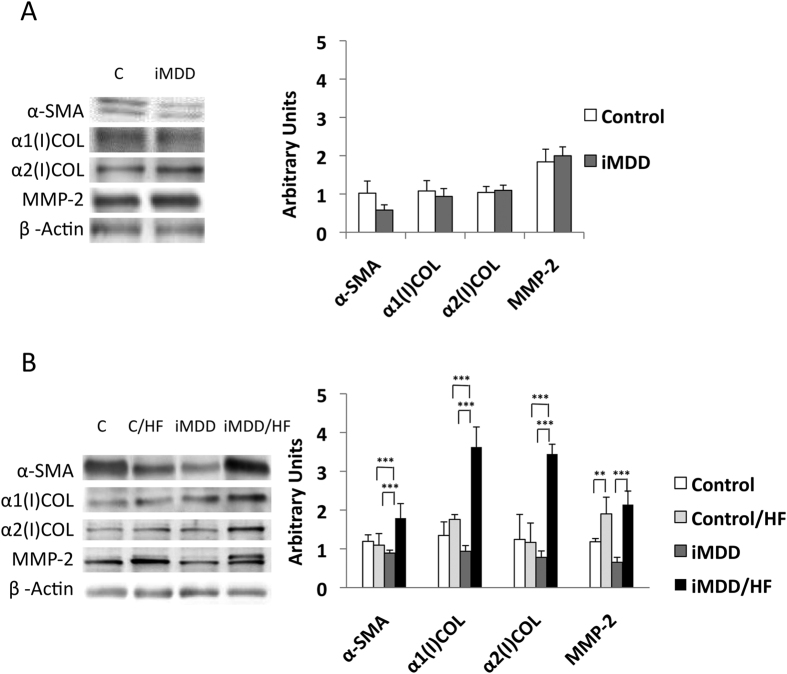
Markers of liver fibrosis. D50 rats were born from dams fed a methyl donor deficient diet (iMDD) diet during pregnancy and lactation and subsequently subjected to high fat (HF) diet between D50 and D185. (**A)** The protein expression of α-SMA (Smooth Muscle Actin), α1(I) collagen, α2(I) collagen and MMP-2 was similar in the liver of D50 iMDD and control animals. (**B**) In contrast, a higher protein expression of α-SMA, α1(I) collagen, α2(I) collagen caspase-1 and MMP-2 was reported in liver from D185 iMDD/HF, compared with other groups. The protein bands were quantified densitometrically, normalized with β-Actin and expressed as arbitrary units; n = 8 in each group, means ± SD, *p < 0.05, **p < 0.01, ***p < 0.001.

Dams were subjected either to an MDD diet (iMDD) or a control diet during pregnancy and lactation and pups received a normal diet after weaning, between D21 and D50. The respective weight of Controls and MDD mothers at D14 of gestation was 214.7 ± 5.5 g and 149.9 ± 7.1 g, (p = 0.0034). We reported 14.4 ± 1.3 and 7.3 ± 1.6 pups in Controls and MDD litters, respectively (p = 0.0150) and a sex ratio (males vs females) of 1.66 and 0.70, respectively (p = 0.0165). We found no litter effect on size and weight of pups from each group. The iMDD rats had no change of body weight/liver weight ratio, compared to control animals (Fig. 1A). In contrast, they had a central obesity reflected by an increased ratio abdominal/body fat, in DEXA examination (Fig. 1A,C). Plasma concentration of homocysteine was lower and ASAT/ALAT ratio was higher in iMDD rats at D50, compared to control animals (Fig. 2A,B). MMA, triglycerides, cholesterol and free fatty acids concentration were similar to those of control animals (Fig. 2A). Histological examination of liver tissues stained by HES, Masson’s trichrome and Sirius Red showed no difference between iMDD and controls and absence of steatosis (Fig. 3A). Taken together, the increase of abdominal fat and ASAT/ALAT ratio was consistent with a foetal programming effect of MDD during pregnancy and lactation, in iMDD rats subjected to a normal diet between D21 and D50. This led us to investigate whether this foetal programming effect could subsequently aggravate the consequences of a high fat diet in the liver of iMDD rats, between D50 and D185. The four groups, control, control/HF, iMDD and iMDD/HF had a similar body weight/liver weight ratio. The control/HF and iMDD/HF had an increased ratio of abdominal/body fat determined by DEXA examination compared to their respective control groups (Fig. 1C). The HF diet did not increase the blood concentration of triglycerides and cholesterol of iMDD/HF rats. In contrast, it produced a 6-fold increase of ASAT/ALAT ratio (Fig. 2B), an increased concentration of free fatty acids (Fig. 2C) and an increase of SAM/SAH ratio (6.5 ± 0.7 versus 4.1 ± 0.3, p < 0.01). Histological analysis of the livers from iMDD/HF rats showed a much more dramatic steatosis, compared to control/HF and iMDD, with a high density of lipid droplets in the cytoplasm of hepatocytes (Fig. 3B). The steatosis was accompanied by a slight fibrosis only in iMDD/HF rats, in histological examination of liver tissues stained by Masson’s trichrome (Fig. 3B). Consistently, the liver of iMDD/HF rats expressed higher protein level of α1(I)collagen, α2(I)collagen and α-SMA, compared to iMDD rats and control/HF rats (Fig. 4A,B). Taken together these data showed that HF produced hallmarks of NASH in iMDD/HF rats, which were not observed in D50 iMDD rats, D50 control and D 185 control/HF animals. This led us to investigate the mechanisms related to NASH, including the expression of genes involved in metabolism dysregulation, inflammation, remodeling and fibrosis.

### iMDD/HF rats had insulin resistance and impaired expression of genes involved in metabolism regulation

**Figure 5:**
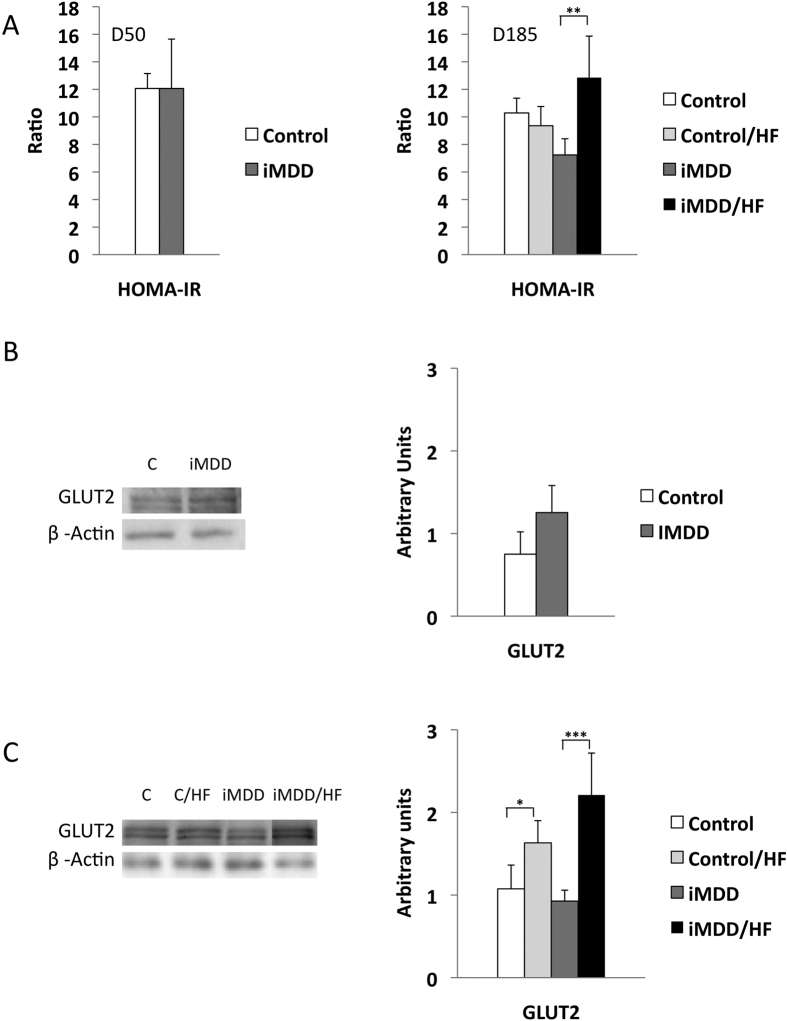
Markers of insulin resistance. D50 rats were born from dams fed a methyl donor deficient diet (iMDD) diet during pregnancy and lactation and subsequently subjected to high fat (HF) diet between D50 and D185. (**A**) HOMA-IR was similar in D50 iMDD rats and increased in D185 iMDD/HF rats, compared to their respective control groups. (**B**) Glut 2 protein expression was similar in D50 iMDD and control animals (**C**) In contrast, protein expression of Glut 2 was increased in control/HF and iMDD/HF, compared to control and iMDD groups, respectively. The protein bands were quantified densitometrically, normalized with β-Actin and expressed as arbitrary units (right); examples of illustrative western blots are showed (left); n = 8 in each group, means ± SD, *p < 0.05, **p < 0.01, ***p < 0.001.

**Figure 6:**
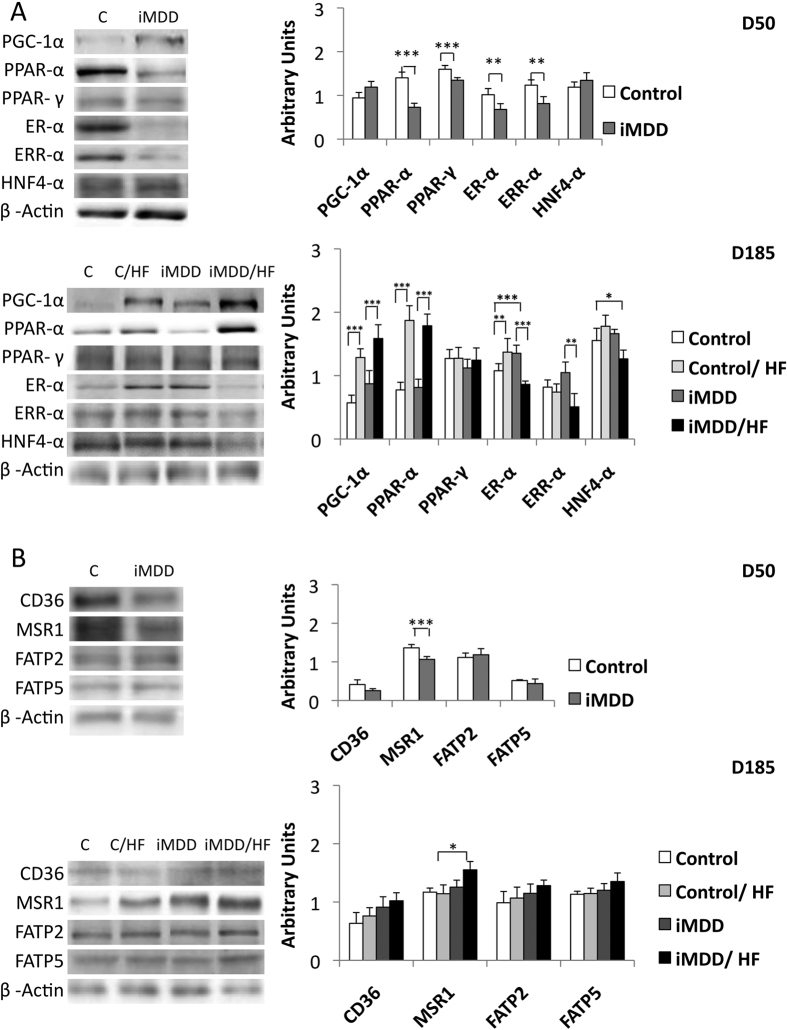
Protein expression of genes related to fatty acid metabolism. D50 rats were born from dams fed a methyl donor deficient diet (iMDD) diet during pregnancy and lactation and subsequently subjected to high fat (HF) diet between D50 and D185. (**A**) ER-α, ERR-α PPAR-α and PPAR-γ had lower protein expression in iMMD that in control D50 rats. In contrast ER-αnand HNF-4α were decreased in iMDD/HF rats, compared to control/HF animals. (**B**) MSR1 has an increased protein expression in iMMD/HF rats, compared to their respective controls. The same trend was observed for CD36, FATP2 and FATP5, but without significance. The protein bands were quantified densitometrically, normalized with β-Actin and expressed as arbitrary units (right); examples of illustrative western blots are showed (left); n = 8 in each group, means ± SD, *p < 0.05, **p < 0.01, ***p < 0.001.

The iMDD/HF animals had a 1.8-fold and 1.4-fold increase of HOMA-IR, when comparing them to iMDD and D185 control/HF rats, respectively (Fig. 5A). Consistently, Glut 2 protein expression of iMDD/HF animals was increased in a much greater extent than in control/HF rats, when comparing the two groups to their respective control groups (Fig. 5B,C). We observed a decreased protein expression of ER-α, ERR-α and PPAR-α in D50 iMMD rats (Fig. 6A). The subsequent exposure to HF diet produced an increased expression of PGC1-α and PPAR-α and a decreased protein expression of ER-α and HNF-4α in control/HF and iMDD/HF rats, suggesting an adaptive regulation of energy metabolism to HF diet (Fig. 6A). We subsequently investigated the expression of proteins involved in liver import of free fatty acids. MSR1 was significantly increased compared to other groups. A same trend was observed for CD36, FATP2 and FATP5, but these changes did not reach significance (Fig. 6B).

### Liver fibrosis was related to activation of inflammasome and fibrogenic pathways in RT-PCR array analysis of iMDD/HF liver rats

**Figure 7:**
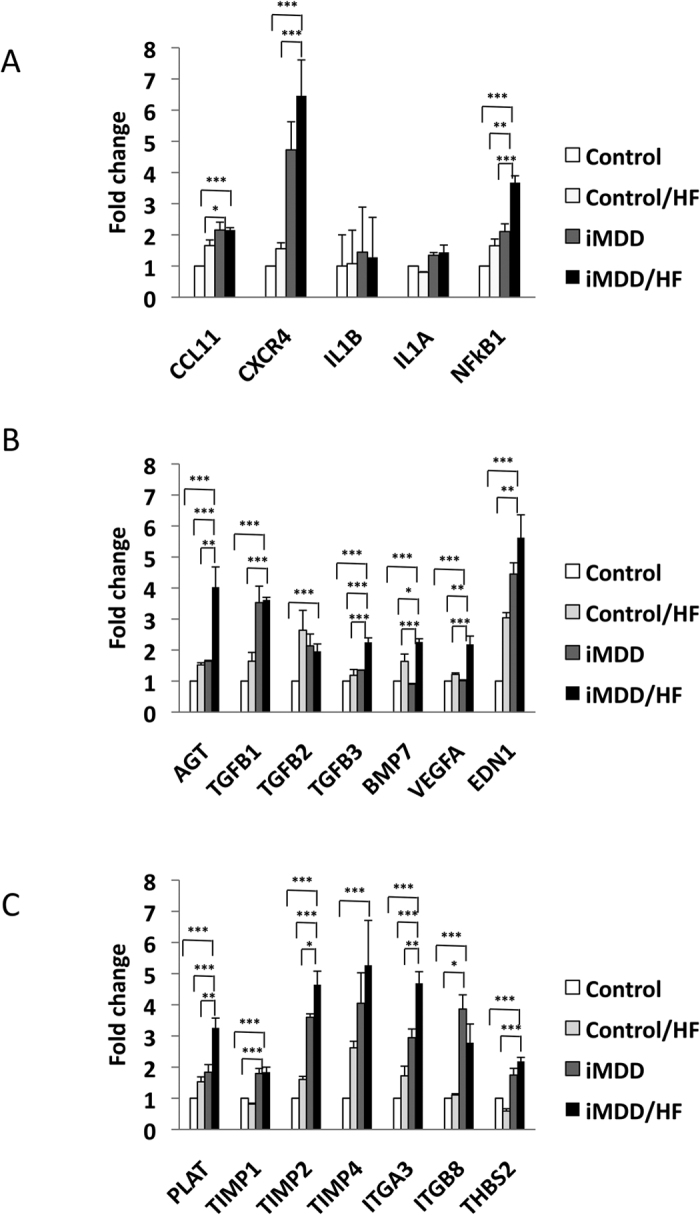
Quantitative analysis of the transcripts from genes related to inflammation, fibrogenic and remodelling pathways. D50 rats were born from dams fed a methyl donor deficient diet (iMDD) diet during pregnancy and lactation and subsequently subjected to high fat (HF) diet between D50 and D185. (**A**) The predominant changes (fold change higher than 2) were observed with *Ccl11*, *Cxcr4, Il1b, Il1a* and *Nfkb1* for inflammatory genes. (**B**) *Agt* (angiotensinogen), *Tgfb1*, *Tgfb2*, *Tgfb3* and *Bmp7* (transforming growth factor beta super family), *Vegfa* (Vascular endothelial growth factor A) and *Edn1* (endothelin 1) for genes involved in stellate cell activation. (**C**) *Plat* (plasminogen activator), *Timp1, Timp2* and *Timp4* (Tissue inhibitor of metalloproteases), *Itga3* and *Itgb8* (integrins) and *Thbs2* (thrombospondin 2) for genes involved in extracellular matrix and cell adhesion; n = 4 in each group, means ± SD, *p < 0.05, **p < 0.01, ***p < 0.001.

We used a RT-qPCR array to perform the quantitative analysis of transcripts from genes related to inflammation, fibrosis and remodelling pathways. Gene expression in liver tissue from iMDD/HF rats was compared to that in control, control/HF and iMDD animals. The predominant changes (fold change higher than 2) were observed with *Ccl11*, *Cxcr4, Il1b* and *Il1a* among proinflammatory genes, *Nfkb1* among the transregulators of inflammation, *Agt* (angiotensinogen), *Agtr1*(angiotensin receptor type 1), *Tgfb1*, *Tgfb2*, *Tgfb3* and *Bmp7* (transforming growth factor beta super family) and *Edn1* (endothelin 1) among genes involved in stellate cell activation, *Akt1* as a signalling protein of *Tgfb* pathways in epithelial-to-mesenchymal cell transition, *Acta2* (SMA) and *Col1a2* among profibrotic genes, *Plat* (plasminogen activator), *Timp1* and *Timp2* (Tissue inhibitor of metalloproteases), *Itga3* and *Itgb8* (integrins) and *Thbs2* (thrombospondin 2) among genes involved in extracellular matrix and cell adhesion (Fig. 7A–C and Supplementary Table S1). These genes have a high level of interactions, including co-expression, co-localization and they are involved in common pathways triggered by angiotensin (Supplementary Table S1). All together, these data suggested an influence of iMDD/HF on the components of NASH pathomechanisms, including inflammation, stellate cell stimulation and subsequent fibrosis and remodelling, through activation of inflammasome, transforming growth factor beta super family and renin-angiotensin system (Supplementary Table S1). We investigated further whether the changes in the main components of these pathways were confirmed at the protein expression level.

### Activation of fibrogenic pathways was related to inflammasome and angiotensinogen/angiotensin receptor, type 1 (AT1) in liver from iMDD/HF rats

**Figure 8:**
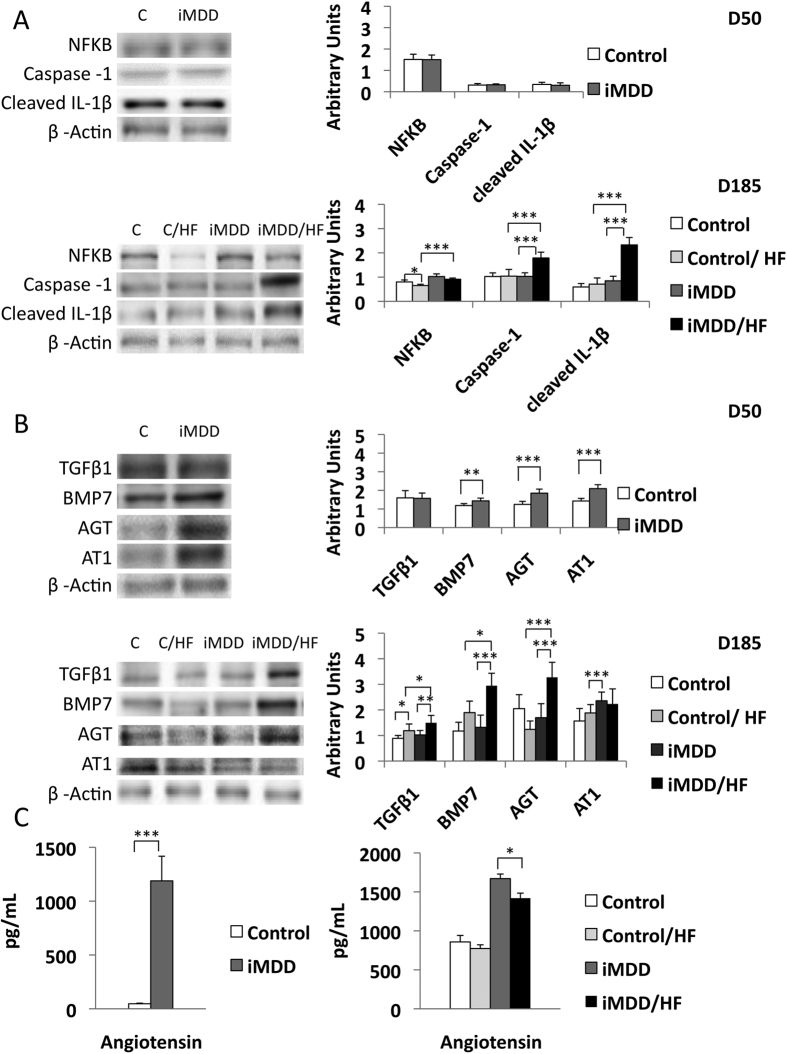
Protein expression of gene related to inflammasome and fibrogenic pathways. D50 rats were born from dams fed a methyl donor deficient diet (iMDD) diet during pregnancy and lactation and subsequently subjected to high fat (HF) diet between D50 and D185. (**A**) expression of NFκB, Caspase 1 and cleaved IL-1β was similar in iMDD and increased in iMDD/HF rats, compared to their respective control groups. (**B**) Angiotensinogen (AGT), angiotensin receptor, type 1 (AT1) and BMP7, but not TGFβ1, were increased in the D50 iMDD animals, while TGFβ1, angiotensin and BMP7 were increased in the D185 iMDD/HF animals, compared to their respective control groups. (**C**) Blood concentration of angiotensin was increased in D50 iMDD rats and D185 iMDD/HF, compared to their respective control groups. The protein bands were quantified densitometrically, normalized with β-Actin and expressed as arbitrary units (right); examples of illustrative western blots are showed (left); n = 8 in each group, means ± SD, *p < 0.05, **p < 0.01, ***p < 0.001.

We observed an increased protein expression of NFκB in iMDD and iMDD/HF rats and a dramatic increase of the protein markers of inflammasome, Caspase 1 and cleaved IL-1β in iMDD/HF rats, compared to the 2 control groups (Fig. 8A). The expression of angiotensinogen, angiotensin receptor, type 1 (AT1) and BMP7, but not TGFβ1, were increased in the D50 iMDD animals (Fig. 8B). In agreement with the RT-PCR data, we confirmed the increased expression of angiotensin, TGFβ1 and BMP7 in the D185 iMDD/HF animals, compared to D185 control, control/HF and iMDD animals (Fig. 8B). We found also an increased blood concentration of angiotensin in D50 iMDD rats and in D185 iMDD/HF (Fig. 8C). These data are consistent with the critical role of angiotensin in fibrogenic pathways of NASH by stimulation of TGFβ1 expression^15^.

## Discussion

Compared to the huge number of epidemiological and experimental studies on overnutrition in the generation of metabolic syndrome, type-2 diabetes and NASH, little attention has been paid to the foetal programming effect of micronutrients restriction on these diseases. Five to 15% of pregnant women have a deficit in folate and/or Cbl, which can lead to fetal programming manifestations^3^. Subsequent over nutrition is becoming epidemic in childhood. Our study was therefore designed to evaluate the links and pathomechanisms between foetal programming and NASH according to these two pre-natal and post-natal dietary windows, in an MDD experimental rat model of foetal programming.

We observed a weight difference between control and MDD dams at D14 of pregnancy. This difference was the consequence of the restriction diet initiated one month before mating, as observed with other restriction diets used to produce rat models of foetal programming^3^. As expected the normal diet between D21 and D50 erased most of the metabolic changes and the liver steatosis of iMDD D21 pups^10^,^16^,^17^. The prominent abnormalities of D50 animals were the permanence of the decreased weight observed previously at birth and D21^10^,^16^,^17^ and the increased proportion of abdominal fat. These abnormalities suggested a higher predisposition of D50 MDD rats to metabolic syndrome outcomes. To assess this hypothesis, we subjected the animals to a HF diet, which had a more limited fat content (50%) than other HF diets currently used in NASH animal models. This HF diet produced mild metabolic and histological abnormalities, but no increase of protein markers of inflammasome, in control/HF rats.

The synergic influence of iMDD programming and HF diet produced hallmarks of NASH, with steatosis, inflammation and fibrosis in liver, compared to control/HF. We found previously that the manifestations of MDD foetal programming, including central abdominal fat and liver and myocardium steatosis resulted from impaired fatty acid beta-oxidation and energy metabolism through the dyregulation of PGC1α co-activation of nuclear receptors, in pups from MDD rats^9^,^10^. These findings are consistent with the increased abdominal fat and ASAT/ALAT ratio observed in iMMD rats. The higher serum concentration of fatty acids and the increased expression of MSR1 produced by HF could favour the import of fatty acids^18^. The liver inflammation was evidenced by the increased protein expression of NFκB and protein markers of inflammasome, Caspase 1 and cleaved IL-1β, in iMDD/HF rats. The greater activation of NFκB has been previously observed in obese patients with NASH^19^, in obese (ob/ob) mice^20^ and in a NASH model of rodents fed a diet deficient in methionine-choline^21^. Caspase-1 and IL-1β play also a master role in NASH of rodents subjected to methionine and choline deficient diet^22^ or high fat diet^23^. In these models, it has been showed that free fatty acids activate NFκB pathway and leads to cytokine production.

Beside the downstream activation of IL-1β in inflammasome, the members of transforming growth factor beta super family play also a master role in stellate cell stimulation and subsequent fibrosis^24^. In consistence with this role, we found an increased transcription expression of the members of this super family, *Tgfb1*, *Tgfb2*, *Tgfb3* and *Bmp7*, in the liver tissue of the iMDD/HF group, compared to other groups. TGFβ1 activated hepatic stellate cells and their transformation into myofibroblasts, as reflected by the increased expression of α-SMA in iMDD/HF rats (Fig. 4B)^25^. The activation of hepatic stellate cells was also consistent with the increased expression of genes of inflammatory chemokines, *Ccl11*, *Cxcr4* and the increased gene expression of integrins (Fig. 7)^24^. Although the critical role of TGF-ß1 in hepatic fibrogenesis is well established, the potential role of BMP7 is less clear. BMP7-stimulated HSCs have an increased expression of type I collagen and fibronectin^26^. Conversely, BMP-7 can attenuate and even prevent the level of hepatic fibrosis in rats through inhibition of the expression of TGF-β1^27^. This preventing effect was limited in regard to the increased fibrosis, in iMDD/HF rats.

Insulin resistance is strongly associated to the pathogenesis of NASH. It was evidenced by increased HOMA-IR and increased GLUT2 expression, in iMDD/HF rats, but not in the D50 iMDD and control/HF rats. It was therefore produced by the two consecutive hits, the foetal programming effect of iMDD and the subsequent exposure of predisposed animals to HF. GLUT2 expression is required for the physiological control of glucose-sensitive genes, and its inactivation in the liver leads to impaired glucose-stimulated insulin secretion through a liver-beta cell axis, which is likely to control beta cell secretion capacity^28^.

*Agt* is one of the master genes involved in the pathogenesis of NASH. The dysregulation of the renin-angiotensin system is known to produce and/or favour most of the changes, which were observed in the iMDD/HF rats, including inflammation, increased expression of TGF-β1 and related markers of fibrosis, insulin resistance and central obesity. The foetal programming effects of iMDD produced synergic effects on the renin-angiotensin system in the liver. The liver expression of *Agt* and the angiotensin blood concentration were dramatically increased in D50 and D185 iMDD rats. In addition, we found an increase of protein expression of AT1 receptor, which is thought to be responsible for most of the adverse effects of angiotensin. These effects were not related to the effect of HF diet only, since they were observed in iMDD/HF but not in control/HF rats. Angiotensin is synthesized by activated hepatic stellate cells^29^. Furthermore, it improves the contraction and proliferation of myofibroblasts and promotes the release of inflammatory and pro-fibrotic cytokines, including TGF-β1, and the deposition of extracellular matrix^30^. The activation of NFκB inflammatory pathway by angiotensin plays also a critical role in insulin resistance of rats subjected to HF^31^. The deleterious angiotensin-AT1 axis impairs glucose homeostasis and favours insulin resistance. Its activation triggers liver inflammation and fibrosis through NFκB and TGF-β1-dependent pathways, as observed in the liver tissue of iMDD/HF rats. The increased angiotensin/AT1 pathway is also consistent with the increased abdominal fat of iMDD/HF rats through its multi-facet influence on visceral metabolism. It modulates adipogenesis, lipogenesis, and white adipose tissue content of the body^32^. Taken together, our data suggest therefore that the dysregulation of renin-angiotensin system played a key role in the fœtal programming effects of MDD on liver inflammation and fibrosis and insulin resistance. However, this system is only one piece of the complex puzzle of the pathways that trigger NASH, considering the contrasted effects of angiotensin inhibitors in NASH animal models^15^,^23^ and their inefficacy in recent clinical trials^33^.

Our model provides new clues in the observations made in populations from India and Nepal, where the prevalence of B12 deficiency is very high during pregnancy^4^,^5^,^6^. It has been showed that this deficiency produces foetal programming effects, which are very similar to those observed in our model, including small birth weight, increased abdominal fat and subsequent insulin resistance in childhood^5^. Children and adolescents are increasingly exposed to high fat intake in western countries and countries with emerging economies. In these populations, the subsequent exposure to high fat diet could be the secondary hit, which participates to the increased prevalence of insulin resistance observed in children born from mothers who had a B12 deficit during pregnancy. Our data suggest that the foetal programming effect of MDD could be one of the early conditions, which trigger the subsequent risk of NASH in obese patients. This hypothesis should deserve further attention in population studies.

## Conclusions

MDD during gestation and lactation produced NASH in adult animals subjected to high fat diet, despite the recovery of a normal histological and metabolic presentation by a control diet between weaning and D50. Insulin resistance and increased expression of angiotensin/AT1 and TGFβ1 were major changes related to inflammasome activation, increased NFκB, activation of stellate cells and increased fibrosis. These results suggest that foetal programming produced by iMDD is a risk factor of NASH. They should be considered in the study of populations, in which the deficit in B12 and/or folate during pregnancy and lactation is frequent.

## Methods

### Animal Treatments

Animal experiments were performed on Wistar rats (Charles River, l’Arbresle, France) and were conducted in accordance with the National Institutes of Health Guide for the Care and Use of Laboratory Animals, in an accredited establishment (Inserm U 954), according to governmental guidelines N86/609/CEE. The animal study has been registered and approved in the French Ministry of higher education and research under the reference APAFIS#1498-2015082015582707vl and has been accepted by the Ethical Committee of Lorraine (N° 66), France. Adult female rats were maintained under standard laboratory conditions, on a 12-hour light/dark cycle, with food and water available ad libitum. One month before pregnancy, animals (n = 8 in each control and MDD group) were fed with either a standard diet (with 16% proteins and 60% nitrogen free extract, caloric intake: 2 800 kcal/kg) or the same diet lacking vitamin B12 (0.0 mg/kg) and folate (0.04 mg/kg) (Special Diet Service, Saint-Gratien, France). The diet provides choline and methionine (220 mg/kg and 0.4% of total content respectively). We equilibrated the number of pups in both groups by sacrificing pups in excess in the control group. The assigned diet was constantly maintained until weaning of the offspring (i.e., postnatal day 21). Pups (16 male pups per group, 2 pups per litter) were fed a normal diet after weaning, from day (D) 21 to D50 and half of them were subsequently exposed to a HF diet between D50 and D185. Ingredients included starch: 113 g/kg, sucrose: 225 g/kg, margarine/oil (50/50): 263 g/kg, casein: 209 g/kg, sweet condensed milk: 100 g/kg, salts: 40 g/kg, vitamins: 10 g/kg, cellulose: 40 g/kg. Carbohydrates supplied 32% of energy, fat, 50% and proteins, 18%. The animals were euthanized at D50 and D185, respectively. This protocol permitted to define 4 study groups, control conditions with normal diet in dams and pups (control), methyl donor deficiency in dams and control diet in pups (iMDD), control diet in dams and high fat in pups (control/HF) and MDD diet in dams and high fat in pups (iMDD/HF), with 6 males per groups. Rats were sacrificed by decapitation after exposure to halothane and the liver were rapidly removed. At the same time, we examined the weight of the body and liver. Immediately after sacrifice, intracardiac blood samples were collected and centrifuged for 10 min at 3000 rpm. Aliquots of serum heparin plasma were stored frozen at −80 °C until analysis. The liver was rapidly collected, washed in PBS1X (2.7 mmol/L KCl, 140 mmol/L NaCl, 6.8 mmol/L Na2HPO4·2H2O, 1.5 mmol/L KH2PO4, pH 7.4), frozen in liquid nitrogen and stored at −80 °C until protein and RNA analysis. The tissue samples used for histological studies were fixed in formol and included in paraffin.

### Whole body composition using DEXA analysis

DEXA analysis was achieved using a QDR-4500A densitometer (Hologic Inc., Waltham, MA, USA) with a module for small animal measurements. The software identifies and estimates global lean mass (g), global fat mass (g), global percentage of fat (%), global mass (g). Rats were anesthetized with an inhalational anaesthesia with 1.5–3 Vol% isoflurane (Forene^®^ 100% (V/V), Abbott, Wiesbaden, Germany) at maintenance, terminated after 40 minutes. After anesthesia, rats were positioned ventrally. Five repeated measurements were performed.

### Biochemical analyses

Plasma concentrations of vitamin B12 and folate were determined by radio-dilution isotope assay (simulTRAC-SNB, ICN, Costa Mesa, USA). Homocysteine, Methylmalonic acid, Succinic acid, SAM and SAH concentrations were measured in plasma by High Performance Liquid Chromatography (Waters, St Quentin, France) coupled to mass spectrometry (Api 4000 Qtrap Applied Biosystems, Courtabœuf, France), as described previously^34^. Insulin was assayed by radioimmunoassay (MP, Biomedicals, Solon, OH, USA). HOMA-IR was calculated using the formula [fasting plasma glucose (mmol/L) × fasting plasma insulin (μIU/mL)]/22.5. Lipids, glycemia, ASAT, ALAT and other routine biochemical parameters were determined in plasma as described previously^10^.

### Histopathological analysis

Histological examination of Hematoxylin-eosin, Masson’s trichrome (Sigma) and Sirius Red stained tissues were performed as described previously^17^. Histological analysis was performed using semi-quantitative items focusing on necroinflammatory lesions, fibrosis and steatosis^17^.

### Real-time quantitative RT-PCR

Rneasy plus Mini Kit (Qiagen, Paris, France) was used for the total RNA extraction by including treatment with DNase. Reverse transcription was done by using the RT2 First Strand Kit (Qiagen, Paris, France). Quantitative RT-PCR was performed on RT2 Profiler PCR Array Rat Fibrosis using RT2 SYBR Green Mastermix kit from Qiagen and reactions were run on the Step One Plus (Applied Biosystems, Saint Aubin, France) using a two-step standard protocol: activation of HotStart DNA Taq Polymerase at 95 °C/10 min and PCR was performed in 40 cycles (one cycle = denaturation at 95 °C/15 s, annealing and extension at 60 °C/1 min). β-actin was used as internal standard, and differences in expression were calculated using the following formula:


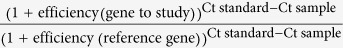


To check for possible DNA contamination of the RNA samples, reactions were also performed in control condition without Omniscript RT enzyme (Qiagen, Courtaboeuf, France).

### Western blot analyses

The extraction of total proteins from the liver was completed by lysing homogenized tissue in RIPA buffer (sodium phosphate anhydrous dibasic, potassium dihydrogen phosphate, 150 mM NaCl, 1% Nonidet P40, 0.5% sodium deoxycholate, sodium dodecyl sulfate and Complete Protease Inhibitors (Roche, Boulogne-Billancourt, France) after washing twice with ice-cold 1X PBS) and then quantified using the bicinchoninic acid assay method. A total of 15–30 μg of protein (30 μg for the analyses of nuclear receptors at D50 and 15 μg for all other analyses) were mixed with an equal volume of 2x Laemmli buffer, denatured by heating the mixture for 5 min at 100 °C, and then resolved by 12% SDS-PAGE. After electrophoretic separation, proteins were transferred to a membrane (polyvinylidene fluoride (PVDF) or nitrocellulose) as described previously^10^,^35^. The proteins were pictured using an ECL detection kit (Amersham, Velizy-Villacoublay, France) and bands were measured by densitometry using Image J 5.1 program. β-Actine was used as an internal reference control, according to our previous evaluations of other proteins, including tubulin and GAPDH^10^.

### Statistical Analyses

Statistical analysis was performed using Statview V.5 software for Windows (SAS Institute, Berkley, California, USA). Continuous variables from RT-qPCR and densitometry analyses of western blots were reported as mean ± S.D. Raw data were compared using the one-way analysis of variance (ANOVA) with Fisher test. The level of significance was set at p-value < 0.05. Results were indicated by asterisks in figures (*p-value < 0.05; **p-value < 0.01 and ***p-value < 0.001).

## Additional Information

**How to cite this article**: Bison, A. *et al.* Foetal programming by methyl donor deficiency produces steato-hepatitis in rats exposed to high fat diet. *Sci. Rep.* **6**, 37207; doi: 10.1038/srep37207 (2016).

**Publisher’s note**: Springer Nature remains neutral with regard to jurisdictional claims in published maps and institutional affiliations.

## Supplementary Material

Supplementary Information
