# Supplementary material for: Foetal programming by methyl donor deficiency produces steato-hepatitis in rats exposed to high fat diet
Source: Sci Rep. 2016 Nov 17;6:37207. doi: 10.1038/srep37207 (PMC5112564; doi:10.1038/srep37207)
Supplement: Supplementary Information [file srep37207-s1.doc]

**Supplementary information**

**Foetal programming by methyl donor deficiency produces steato-hepatitis in rats exposed to high fat diet**

**Running Head: Fetal programming of steato-hepatitis**

Anaïs Bison^1^, Aude Bressenot^1^, Zhen Li^1^, Ilef Elamouri^1^, Eva Feigerlova^1^, Lu Peng^1^, Remi Houlgatte^1^, Bernard Beck^1^, Gregory Pourié^1^, Jean-Marc Alberto^1^, Remy Umoret^1^, Guillaume Conroy^1^, Jean-Pierre Bronowicki^1^, Jean-Louis Guéant^1*†^, Rosa-Maria Guéant-Rodriguez^1*†^

^1^ Inserm U954, Nutrition-Genetics-Environmental Risk Exposure (N-GERE), University of Lorraine, BP 184, 54511, Vandœuvre-lès-Nancy, France

*Corresponding authors.

† Equal contribution

***Correspondence*:** † Rosa Maria Guéant-Rodriguez, MD, PhD, and Jean-Louis Guéant, MD, DSc, Inserm U-954, Faculté de Médecine, 9 avenue de la Forêt de Haye, B.P. 184, 54500, Nancy-Vandœuvre, France. Phone: (33) 3 83 68 39 92. Fax: (33) 3 83 68 32 79. E-mail: [rosa-maria.gueant-rodriguez@univ-lorraine.fr](mailto:rosa-maria.gueant-rodriguez@univ-lorraine.fr) and [jean-louis.gueant@medecine.uhp-nancy.fr](mailto:jean-louis.gueant@medecine.uhpchu-nancy.fr)

Supplementary Table 1 : Quantitative analysis of transcripts from genes related to inflammation, fibrosis and remodelling pathways.

| **Gene name** | **Gene name** | **Function** | **Control** | **Control/HF** | **iMDD** | **iMDD/HF** | **iMDD/HF vs contol** | **iMDD/HF vs c/HF** | **iMDD/HF vs iMDD** |
| --- | --- | --- | --- | --- | --- | --- | --- | --- | --- |
| acta2 | Smooth muscle alpha-actin | Pro-fibrotic | 1.0 ± 0.0 | 1.2 ± 0.0 | 2.7 ± 0.0 | 2.6 ± 0.1 | <0.0001 | <0.0001 | 0.4750 |
| agt | Angiotensinogen | Pro-fibrotic | 1.0 ± 0.0 | 1.5 ± 0.1 | 1.6 ± 0.0 | 4.0 ± 0.7 | 0.0002 | <0.0001 | 0.0018 |
| akt1 | V-akt murine thymoma viral oncogene homolog 1 | Epithelial to mesenchymal transition | 1.0 ± 0.0 | 1.1 ± 0.0 | 1.7 ± 0.1 | 3.8 ± 0.6 | 0.0002 | 0.0002 | 0.0028 |
| bcl2 | B-cell CLL/lymphoma 2 | Fibrosis | 1.0 ± 0.0 | 1.9 ± 0.2 | 2.9 ± 0.5 | 2.4 ± 0.5 | 0.0195 | 0.4329 | 0.5106 |
| bmp7 | Bone morphogenetic protein 7 | Anti-fibrotic | 1.0 ± 0.0 | 1.6 ± 0.2 | 0.9 ± 0.0 | 2.3 ± 0.1 | <0.0001 | 0.0255 | <0.0001 |
| cav1 | Caveolin 1, caveolae protein | Signal traduction | 1.0 ± 0.0 | 3.6 ± 1.1 | 6.4 ± 1.6 | 5.9 ± 1.3 | 0.0017 | 0.2050 | 0.8156 |
| ccl11 | Chemokine (C-C motif) ligand 11 | Inflammatory chemokine | 1.0 ± 0.0 | 1.7 ± 0.2 | 2.2 ±0.3 | 2.2 ± 0.1 | <0.0001 | 0.0204 | 0.9999 |
| cxcr4 | Chemokine (C-X-C motif) receptor 4 | Inflammatory chemokine | 1.0 ± 0.0 | 1.6 ± 0.2 | 4.7 ± 0.9 | 6.5 ± 1.2 | 0.0002 | 0.0005 | 0.2506 |
| edn1 | Endothelin 1 | Growth factor | 1.0 ± 0.0 | 3.0 ± 0.2 | 4.5 ± 0.4 | 5.6 ± 0.7 | <0.0001 | 0.0030 | 0.1690 |
| il1a | Interleukin 1 alpha | Inflammatory cytokine | 1.0 ± 0.0 | 0.8 ± 0.0 | 1.3 ± 0.1 | 1.4 ± 0.2 | 0.0756 | 0.0137 | 0.7078 |
| il1b | Interleukin 1 beta | Inflammatory cytokine | 1.0 ± 0.0 | 1.1 ± 0.1 | 1.4 ± 0.1 | 1.3 ± 0.2 | 0.0908 | 0.2611 | 0.3797 |
| il4 | Interleukin 4 | Pro-fibrotic | 1.0 ± 0.0 | 1.6 ± 0.3 | 1.7 ± 0.5 | 3.5 ± 1.0 | 0.0212 | 0.0948 | 0.1264 |
| itga1 | Integrin, alpha 1 | Cell adhesion molecule | 1.0 ± 0.0 | 1.5 ± 0.0 | 1.3 ± 0.1 | 2.0 ± 0.1 | <0.0001 | 0.0005 | 0.0001 |
| itga2 | Integrin, alpha 2 | Cell adhesion molecule | 1.0 ± 0.0 | 0.9 ± 0.1 | 1.1 ± 0.0 | 0.6 ± 0.1 | 0.0034 | 0.0698 | 0.0004 |
| itga3 | Integrin, alpha 3 | Cell adhesion molecule | 1.0 ± 0.0 | 1.7 ± 0.3 | 2.9 ± 0.3 | 4.7 ± 0.4 | <0.0001 | <0.0001 | 0.0015 |
| itgb6 | Integrin, beta 6 | Cell adhesion molecule | 1.0 ± 0.0 | 3.3 ± 0.8 | 5.5 ± 1.8 | 3.4 ± 0.9 | 0.0147 | 0.9194 | 0.3091 |
| itgb8 | Integrin, beta 8 | Cell adhesion molecule | 1.0 ± 0.0 | 1.1 ± 0.8 | 3.9 ± 1.8 | 2.8 ± 0.9 | <0.0001 | 0.0118 | 0.1679 |
| lox | Lysyl oxidase | Extracellular matrix remodeling enzyme | 1.0 ± 0.0 | 2.6 ± 0.2 | 5.9 ± 1.7 | 5.4 ± 0.5 | <0.0001 | 0.0001 | 0.8068 |
| mmp3 | Matrix metallopeptidase 3 | Extracellular matrix remodeling enzyme | 1.0 ± 0.0 | 0.6 ± 0.1 | 0.6 ± 0.1 | 0.4 ± 0.1 | <0.0001 | 0.0935 | 0.0140 |
| mmp14 | Matrix metallopeptidase 14 | Extracellular matrix remodeling enzyme | 1.0 ± 0.0 | 1.3 ± 0.1 | 1.4 ± 0.1 | 2.5 ± 0.4 | 0.0013 | 0.0096 | 0.0140 |
| nfkb1 | Nuclear factor of kappa light polypeptide gene enhancer in B-cells 1 | Transcription factor | 1.0 ± 0.0 | 1.7 ± 0.2 | 2.1 ± 0.2 | 3.7 ± 0.2 | <0.0001 | <0.0001 | 0.0002 |
| pdgfa | Platelet-derived growth factor alpha polypeptide | Growth factor | 1.0 ± 0.0 | 1.1 ± 0.3 | 1.2 ± 0.0 | 3.0 ± 0.4 | 0.0001 | 0.0012 | 0.0003 |
| plat | Plasminogen activator, tissue | ECM remodeling enzyme | 1.0 ± 0.0 | 1.5 ± 0.2 | 1.8 ± 0.2 | 3.3 ± 0.3 | <0.0001 | 0.0001 | 0.0021 |
| serpina1 | Serpin peptidase inhibitor, clade A | ECM remodeling enzymes | 1.0 ± 0.0 | 1.1 ± 0.0 | 1.3 ± 0.1 | 2.1 ± 0.2 | 0.0001 | 0.0003 | 0.0043 |
| serpine1 | Serpin peptidase inhibitor, clade E | ECM remodeling enzymes | 1.0 ± 0.0 | 1.1 ± 0.0 | 1.4 ± 0.1 | 2.6 ± 0.4 | <0.0001 | 0.0141 | 0.0483 |
| serpinh1 | Serine (or cysteine) peptidase inhibitor, clade H, member 1 | ECM remodeling enzymes | 1.0 ± 0.0 | 0.7 ± 0.0 | 1.8 ± 0.1 | 2.8 ± 0.4 | 0.0002 | <0.0001 | 0.0182 |
| smad3 | SMAD family member 3 | Epithelial to mesenchymal transition | 1.0 ± 0.0 | 1.0 ± 0.1 | 1.1 ± 0.1 | 2.7 ± 0.5 | 0.0074 | 0.0077 | 0.0131 |
| smad7 | SMAD family member 7 | Epithelial to mesenchymal transition | 1.0 ± 0.0 | 1.3 ± 0.1 | 1.3 ± 1.2 | 2.4 ± 0.6 | 0.0244 | 0.0730 | 0.0969 |
| stat6 | Signal transducer and activator of transcription 6 | Transcription factor | 1.0 ± 0.0 | 1.1 ± 0.0 | 0.9 ± 0.0 | 2.1 ± 0.2 | <0.0001 | 0.0001 | <0.0001 |
| tgfb1 | Transforming growth factor, beta 1 | Signal traduction | 1.0 ± 0.0 | 1.6 ± 0.3 | 3.5 ± 0.5 | 3.6 ± 0.1 | <0.0001 | <0.0001 | 0.8759 |
| tgfb2 | Transforming growth factor, beta 2 | Signal traduction | 1.0 ± 0.0 | 2.6 ± 0.6 | 2.1 ± 0.4 | 2.0 ± 0.2 | 0.0007 | 0.3322 | 0.7013 |
| tgfb3 | Transforming growth factor, beta 3 | Signal traduction | 1.0 ± 0.0 | 1.2 ± 0.2 | 1.4 ± 0.0 | 2.3 ± 0.1 | <0.0001 | 0.0003 | <0.0001 |
| tgfbr2 | Transforming growth factor, beta receptor II | Signal traduction | 1.0 ± 0.0 | 1.4 ± 0.0 | 1.5 ± 0.1 | 2.7 ± 0.3 | <0.0001 | 0.0005 | 0.0019 |
| thbs2 | Thrombospondin 2 | Signal traduction | 1.0 ± 0.0 | 0.6 ± 0.1 | 1.7 ± 0.2 | 2.2 ± 0.2 | <0.0001 | <0.0001 | 0.0959 |
| timp1 | TIMP metallopeptidase inhibitor 1 | Epithelial to mesenchymal transition | 1.0 ± 0.0 | 0.8 ± 0.0 | 1.8 ± 0.2 | 1.8 ± 0.2 | 0.0001 | <0.0001 | 0.8454 |
| timp2 | TIMP metallopeptidase inhibitor 2 | Epithelial to mesenchymal transition | 1.0 ± 0.0 | 1.6 ± 0.1 | 3.6 ± 0.1 | 4.6 ± 0.4 | <0.0001 | <0.0001 | 0.0317 |
| timp4 | TIMP metallopeptidase inhibitor 4 | Epithelial to mesenchymal transition | 1.0 ± 0.0 | 2.6 ± 0.2 | 4.1 ± 1.0 | 5.3 ± 1.4 | 0.0002 | 0.0846 | 0.4915 |
| vegfa | Vascular endothelial growth factor A | Growth factor | 1.0 ± 0.0 | 1.2 ± 0.0 | 1.0 ± 0.0 | 2.2 ± 0.3 | 0.0003 | 0.0020 | 0.0003 |
